# Molecular Targets in *Campylobacter* Infections

**DOI:** 10.3390/biom13030409

**Published:** 2023-02-22

**Authors:** Markus M. Heimesaat, Steffen Backert, Thomas Alter, Stefan Bereswill

**Affiliations:** 1 Gastrointestinal Microbiology Research Group, Institute of Microbiology, Infectious Diseases and Immunology, Charité—University Medicine Berlin, Corporate Member of Free University Berlin, Humboldt University Berlin, and Berlin Institute of Health, D-12203 Berlin, Germany; 2 Division of Microbiology, Department of Biology, Friedrich-Alexander-Universität Erlangen-Nürnberg, D-91058 Erlangen, Germany; 3 Institute of Food Safety and Food Hygiene, School of Veterinary Medicine, Free University Berlin, D-14163 Berlin, Germany

**Keywords:** one health concept, infection prevention strategies, campylobacteriosis, *Campylobacter jejuni* infection models

## Abstract

Human campylobacteriosis results from foodborne infections with *Campylobacter* bacteria such as *Campylobacter jejuni* and *Campylobacter coli*, and represents a leading cause of bacterial gastroenteritis worldwide. After consumption of contaminated poultry meat, constituting the major source of pathogenic transfer to humans, infected patients develop abdominal pain and diarrhea. Post-infectious disorders following acute enteritis may occur and affect the nervous system, the joints or the intestines. Immunocompromising comorbidities in infected patients favor bacteremia, leading to vascular inflammation and septicemia. Prevention of human infection is achieved by hygiene measures focusing on the reduction of pathogenic food contamination. Molecular targets for the treatment and prevention of campylobacteriosis include bacterial pathogenicity and virulence factors involved in motility, adhesion, invasion, oxygen detoxification, acid resistance and biofilm formation. This repertoire of intervention measures has recently been completed by drugs dampening the pro-inflammatory immune responses induced by the *Campylobacter* endotoxin lipo-oligosaccharide. Novel pharmaceutical strategies will combine anti-pathogenic and anti-inflammatory effects to reduce the risk of both anti-microbial resistance and post-infectious sequelae of acute enteritis. Novel strategies and actual trends in the combat of *Campylobacter* infections are presented in this review, alongside molecular targets applied for prevention and treatment strategies.

## 1. Introduction

Campylobacteriosis is the leading cause of bacterial gastroenteritis worldwide [1,2,3,4,5]. Human campylobacteriosis is mostly caused by *Campylobacter jejuni* and less frequently by *Campylobacter coli*, and occurs predominantly after consumption of contaminated chicken meat [6]. According to a recent EFSA report, a total of 31% of chilled broiler carcasses sampled at slaughterhouses were *Campylobacter*-positive. At the food level, the highest *Campylobacter* proportions were detected in fresh meat from turkeys (12.9%), followed by fresh meat from broilers (11.5%) [7]. The potent pathogens cause acute and severe enteritis in humans, but are harmless commensals in domestic animals. The asymptomatic colonization in chicken flocks supports the formation of huge pathogen reservoirs [8,9]. Thus, reduction of *Campylobacter* contamination in chicken breeding and the associated food chain by hygiene interventions is key to the prevention of human campylobacteriosis. Humans develop enteritis even after ingestion of low pathogen doses in the range of hundreds of living bacteria. Campylobacteriosis symptoms vary from mild abdominal pain to severe, inflammatory, bloody diarrhea associated with grievous cramps and fever, which can last for more than a week (as reviewed elsewhere [10,11,12,13,14]). The severity of initial enteritis is significantly associated with the risk of post-infectious sequelae, which may appear weeks or months after the intestinal infection [15]. Post-infectious sequelae include Guillain–Barré-Syndrome (GBS) [16,17], reactive arthritis [18], inflammatory bowel diseases [19,20,21] or irritable bowel syndrome [18]. In rare cases, *Campylobacter* bacteremia occurring in immunocompromised individuals may lead to meningitis [22] or cardiovascular diseases [23] (for reviews concerning post-infectious complications, see [4,13,24,25,26]). Most importantly, the risk for post-infectious sequelae increases with the severity of the initial enteritis [14]. This supports the need for novel prevention and treatment strategies, given that most patients receive rehydration measures, but no causative treatment of the inflammation [14,17,27]. In consequence of the socioeconomic burden caused by campylobacteriosis, industrialized countries have established control measures by reporting and undertaking statistical analysis of *Campylobacter*-associated diseases. However, unreported campylobacteriosis cases are expected to exceed by far the number of cases reported in published statistics [28,29,30].

## 2. From Microbiology and Intestinal Inflammation to Molecular Targeting of Campylobacteriosis

The multifaceted campylobacteriosis symptoms and the severe post-infectious sequelae have stimulated a wealth of research into the pathogenesis of *C. jejuni* infections. While investigations on the inflammation have long been hampered by the lack of experimental murine infection models [31], there is remarkable progress in the discovery of bacterial pathogenicity and virulence factors. Several microbiological characteristics represent valuable molecular targets for intervention strategies to prevent and treat *C. jejuni* infections.

*Campylobacters* belong to the ε-proteobacteria subphylum of Gram-negative bacteria. The spiral *C. jejuni* bacteria are thermophilic with a growth optimum of 42 °C. The chromosome carries around 1600 genes at high density [32,33,34]. The low gene number restricts the metabolic capabilities of *C. jejuni* and supports a fastidious and microaerophilic lifestyle with a limited repertoire for environmental adaptation [32,33,34]. Many regulatory systems and carbohydrate utilization pathways present in other Gram-negative pathogens are absent in *C. jejuni*, as discussed below [30]. This limited adaptation capacity results in sensitivity to environmental stress, favoring the sensitivity of *C. jejuni* to organic acids as a means of preventive hygiene measures in the poultry industries [35]. In humans, the use of proton pump inhibitors increases the risk of *C. jejuni* infection, indicating that gastric acid protects from campylobacteriosis [36]. Therefore, maintaining stomach health is a major goal for the prevention of campylobacteriosis and enteric infections in general.

**Figure 1:**
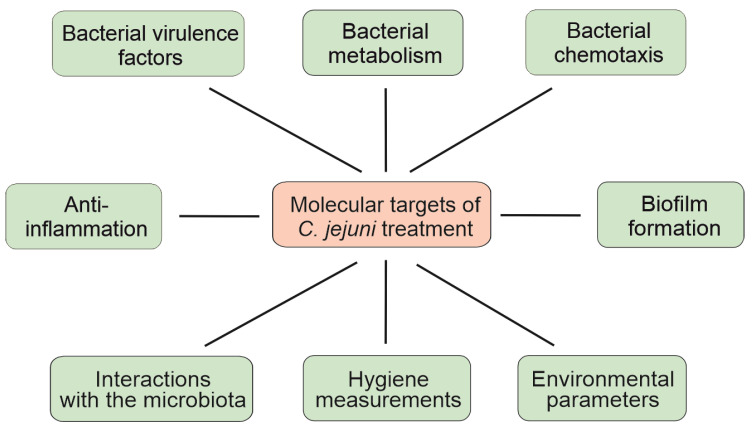
Overview of the molecular targets in *C. jejuni* treatment strategies. For more details, see text.

In human hosts, ingested *C. jejuni* bacteria surviving gastric acid, bile acids and digestive enzymes in the stomach and upper intestines enter the intestinal mucus layer, which is facilitated by their motility. After adhesion to epithelial cells and invasion of the sub-epithelial tissues, the pathogens induce inflammation by activation of the innate immune system. Thus, in the human host, adaptation to acid, bile and oxygen, as well as the structure and functions of flagella, adhesion and invasion proteins, represent major pathogenicity determinants of *C. jejuni* that are essential for the onset, progression and clinical outcome of campylobacteriosis [37,38,39,40]. In line with the mechanisms mediating bacterial survival in the environment, these factors serve as valid molecular targets for prevention and treatment strategies, as is summarized in the following paragraphs and in Figure 1. In contrast, the use of inflammation as a molecular target for the amelioration of campylobacteriosis awaits further investigation, and corresponding developments are described in the last paragraph of this review.

## 3. Pathogenesis of *Campylobacter* Infection and Introduction to the Major Molecular Targets within *C. jejuni*

Our knowledge of mechanisms involved in the molecular pathogenesis of *C. jejuni* is based on clinical studies, investigations of the bacteria in cell culture in vitro and in animal models such as chicken and mice [5,41]. After ingestion, the intruding bacteria move very effectively by using their rotating bipolar flagella. *C. jejuni* controls its motility by a multifaceted chemotaxis machinery that is guided through various chemoreceptors. These chemotaxis signaling pathways allow the pathogen to move toward beneficial chemoattractants and repulse from chemorepellents. The best-characterized *C. jejuni* chemotaxis cascade comprises the CheA/CheW-CheY proteins, all of which are essential for successful infection of human and animal hosts [42,43,44]. This machinery is used by *C. jejuni* to cross the mucous layer of the lower gastrointestinal tract, a nutrient-rich niche that is perfectly suited for bacterial colonization and growth [45]. The catabolic versatility of *C. jejuni* is limited compared to other enterobacteria, as various pathways of the carbohydrate metabolism are absent. However, *C. jejuni* exhibits numerous chemotactic activities, most notably the sensing of amino acids such as asparagine and aspartate, lactate, formate and intermediates of the citric acid cycle, which are the key energy sources for the bacterium [46,47]. Thus, the metabolism of *C. jejuni* is highly specialized, a feature that can be used to target this pathogen. A screen of an inhibitor library comprising about 147,000 compounds revealed a number of small molecules that profoundly compromised the in vitro growth of *C. jejuni* and flagellar gene expression, respectively [48]. Some of these inhibitors were found to display bacteriostatic effects on *C. jejuni*, while not being harmful to host cells. Oral administration of at least one compound resulted in reduced *C. jejuni* loads in a chicken model [48]. In addition, several anti-microbial compounds were found to change the morphology of *C. jejuni*. For instance, meropenem triggered bulking of the bacteria, imipenem induced the conversion of spiral-shaped to coccoid forms, and sitafloxacin resulted in bacterial elongation, all of which were associated with severely compromised *C. jejuni* motility [49]. These studies are very promising with regard to pinpointing anti-*C. jejuni* activities, but further investigations are clearly required to validate the specific cellular targets of the compounds, and their possible application in human therapy.

*C. jejuni* colonizes the intestinal mucus layer as an initial step of the infection [50]. Subsequently, the pathogen adheres to intestinal epithelial cells, transmigrates across the intestinal epithelium and initiates host cell entry and intracellular survival. Interestingly, the addition of purified intestinal chicken mucus, but not mucus of human origin, inhibited both *C. jejuni* binding and entry of human epithelial cells [51]. Bacterial invasion of the host cells was more strongly repressed than *C. jejuni* adhesion. Pre-treatment of chicken mucin with sodium-metaperiodate, a chemical compound that oxidizes glycan entities on mucin glycoproteins, restored *C. jejuni* invasion of human cells [51]. In contrast, exposure of chicken mucin to other enzymes, specifically sialidase or fucosidase, did not abolish *C. jejuni* cell binding and entry. These observations strongly suggest that glycosylation of chicken mucin mediates the observed suppressive effects on *C. jejuni* host cell interactions. However, the exact interaction points and specific glycan residues in the mucin are still unknown and should be explored in future studies.

Besides *C. jejuni* colonization of the mucus layer, the bacteria can also bind to enterocytes, which is facilitated by an array of surface-exposed adhesion proteins. Among several candidates, the two fibronectin-binding proteins CadF and FlpA have been studied in detail [52,53]. These proteins also mediate the invasion of *C. jejuni* into the intestinal epithelium from the bottom of the cells via a basal mechanism using fibronectin and integrin receptors. *C. jejuni* invasion is enhanced by so-called Cia (*Campylobacter* invasion antigens) proteins such as CiaB, CiaC, CiaD and others that are secreted by the flagellar type III secretion system (fT3SS) [54,55,56]. These interactions also trigger the activation of the small Rho GTPases Rac1 and Cdc42, which induce cytoskeletal reorganizations and bacterial uptake [52,53,57,58,59,60]. The ability to survive intracellularly in vacuoles enhances the pathogenicity of *C. jejuni*. Various natural compounds from plant extracts with so-called GRAS (generally recognized as safe) status have been screened with the aim of identifying candidates targeting binding and invasion of host cells by *C. jejuni* during infection. For example, glucuronic-acid-enriched polysaccharides prepared from *Abelmoschus esculentus* (okra fruit) efficiently prevented *C. jejuni* binding to host cells in vitro [61]. In addition, extracts from various citrus plants (*Citrus medica*, *Citrus limon* and *Citrus aurantium*) inhibited adhesion and invasion of *C. jejuni* into cultured HeLa cells and reduced the expression of *ciaB* and *cadF* genes [62]. Other studies have described that peppermint essential oil, a commonly used substance in the treatment of gastrointestinal diseases, led to decreased expression of various *C. jejuni* virulence-associated genes such as *cheY*, *cadF* and the flagellar genes *flhB* and *flgE*, which inhibited bacterial motility [63]. Thus, specific extracts from plants may represent innovative and useful new therapeutic reagents to treat *C. jejuni* infections in future.

In addition, *C. jejuni* secretes the serine protease HtrA into the extracellular milieu [64]. This protease disrupts tight and adherens junctions by cleavage of important junctional proteins such as occludin [65], claudin-8 [66] and E-cadherin [67,68]. This leads to the opening of cell–cell junctions and epithelial barrier dysfunction during infection. *C. jejuni* is thus able to overcome the intestinal epithelial barrier by transmigration via the paracellular route, reaching deeper tissue layers and even migrating to other organs such as liver, spleen or mesenteric lymph nodes, which plays a role in the context of bacteremia and sepsis [40]. It appears that *C. jejuni* HtrA activity also contributes to in vivo pathogenesis in infant mice [69] and interleukin (IL)-10^-/-^ knockout mice [70,71]. Epithelial barrier dysfunction during *C. jejuni* infection disturbed the ion homeostasis in the intestinal epithelium, which leads to the loss of water and diarrhea, called leaky flux phenotype [72]. Several infection-induced immune mediators, such as tumor necrosis factor (TNF), IL-6, IL-8, IL-12, IL-23 and IL-1β, intensify the barrier damage as discussed below. They also intensify diarrhea, as characterized by sodium malabsorption through dysregulation of the sodium channel ENaC [73]. Recent studies in cell culture in vitro and in mice in vivo indicated a significant reduction of *C. jejuni*-triggered epithelial barrier dysfunction by application of the polyphenol curcumin [74] or vitamin D [73]. Vitamin D treatment also decreased *C. jejuni* transmigration across polarized epithelial cells. Thus, curcumin and vitamin D could be promising compounds for the treatment of *C. jejuni* infection in animals and humans. Finally, the development of small molecule inhibitors interfering with HtrA protease activity is another promising novel approach in anti-bacterial therapy [75]. For instance, computer-assisted de novo design revealed a small inhibitor compound interfering with protease HtrA from *Helicobacter pylori*, a gastric pathogen evolutionarily related to *C. jejuni* [76]. Molecular binding and functional activity studies resulted in the identification and characterization of this presently best-in-class HtrA inhibitor. However, while being effective against *H. pylori* HtrA, this compound only marginally reduced the number of transmigrated *C. jejuni*. Nevertheless, these data demonstrated the feasibility of pioneering inhibitor compounds with tailor-made activity, which could be also applied to *C. jejuni* HtrA in the therapy of corresponding infections.

The above-described pathogenic processes of *C. jejuni* are further enhanced by the CDT (cytolethal distending toxin), which is secreted by subpopulations of the pathogen [77]. CDT function depends on the presence of three genes, named *cdtA*, *cdtB* and *cdtC*. When expressed, the protein subunits CdtA, CdtB and CdtC bind to each other and form a tripartite complex that is called active holotoxin. The CdtA and CdtC subunits are required for the delivery of CdtB into the cytoplasm of target cells. CdtB represents the catalytically active subunit, which displays DNase activity and is transferred to the host cell nucleus. This activity results in chromosomal DNA cleavage, nuclear and cellular swelling and G(2)/M cell cycle arrest [77,78]. As described above, innovative new tools for better controlling the *C. jejuni* infection are natural products derived from plants. Among those, extracts of silvery mugwort (*Artemisia ludoviciana*) and sweet acacia (*Acacia farnesiana*) inhibited both the adherence of *C. jejuni* to cultured Vero cells as well as CDT activity [79]. Another report investigated the usefulness of the phytochemicals eugenol, carvacrol and trans-cinnamaldehyde during *C. jejuni* infection of polarized Caco-2 cells [80]. Most of the phytochemicals reduced the expression of CDT and diminished *C. jejuni* motility, cellular adherence and invasion, as well as bacterial transmigration [80]. Thus, these plant extracts and phytochemicals represent additional candidates to be investigated for prevention or treatment of *C. jejuni* contamination in food products, and potential anti-microbial therapy of the infections.

## 4. Environmental Survival Factors Conduct Campylobacteriosis Prevention

*Campylobacter* are highly susceptible to different stress conditions that are encountered by these bacteria in the environment or in the food chain. *Campylobacter* have developed mechanisms to adapt to these conditions, however, enabling them to persist in the environment and the food chain and survive technological stressors applied during food processing. Table 1 summarizes the factors that influence the survival of *Campylobacter* from the farm to the consumer.

A number of studies used genomic and transcriptomic data to investigate mechanisms of stress response and adaption and additionally linked those data to phenotypic assays [110,111,112,113,114]. While the majority of these studies applied a rather mechanistic approach, a recent review focused specifically on the survival and adaptation of *C. jejuni* within the poultry production chain [109]. De Vries et al. (2017) performed a genome-wide fitness analysis by transposon mutant library screening of *C. jejuni*. The authors demonstrated that a large part of the genome is related to fitness and survival, with 486 out of 1424 genes coding for fitness factors [115], for type III and type VI secretion systems [116], for the ferric uptake regulator [114] and furthermore, for components of the flagellar system and cell envelope [117]. Increased aerotolerance may be an important survival mechanism of microaerophilic *Campylobacter* when encountering extra-intestinal environments [118]. Studies showed that aerotolerant or hyper-aerotolerant strains exist that also contain a higher rate of virulence-related genes if compared to aero-sensitive strains [119]. While investigating an aerotolerant *C. jejuni* strain under aerobic conditions, Rodrigues et al. (2016) identified overexpression of proteins related to oxidative stress response, to amino acid and to iron uptake [120]. The authors concluded that differential gene expression patterns contribute to aerotolerance in the investigated strain rather than the presence of unique stress response genes. Low temperature conditions have been shown to induce oxidative stress response [115]. Subsequently, genes related to oxidative stress response are needed for survival at low temperatures, such as oxidoreductase (*trxC*) and regulator of oxidative stress (*perR*). Furthermore, attenuation of *mcp4_2* (involved in chemotaxis), *kefB* and *czcD* (antiporters) and *fabI* (fatty acid metabolism) for survival in different habitats was associated with a response to lower temperatures [115]. *Campylobacter* strains differ significantly in their ability to survive outside their hosts. It is speculated that *Campylobacter* strains that potentially adapted to the environment have improved fitness through the evolution of stress resistance mechanisms [121,122].

Formation of biofilms or integration into existing biofilms increases the survival potential of bacteria in the environment. *Campylobacter* can form biofilms or (rather) integrate into pre-existing biofilms present on contact surfaces or equipment of the food chain, such as slaughter equipment, water supplies or plumbing systems [123]. The ability to form biofilms is lower in *Campylobacter* compared to other bacteria. While monospecies *Campylobacter* biofilms have not been demonstrated in vivo, secondary colonization or integration into pre-existing biofilms is possible and of practical concern [124]. A summary of genes involved in biofilm formation was recently provided by Püning et al. (2021) [125]. Based on data published by Tram et al. (2020), approximately 600 genes are differentially expressed in *Campylobacter* during biofilm formation, with induction of genes related to iron metabolism, cell division and glycan production, among others. Repressed genes are linked to metabolism and parts of the chemotaxis pathway [126]. The ability of flagella-associated attachment influences the degree of biofilm formation [127]. Specifically, biofilm formation depends on flagellar O-linked glycan modification [128]. Other factors influencing the degree of biofilm formation are alkyl hydroperoxide reductase (*ahpC*) and catalase A (*katA*), highlighting the role of oxidative stress under these circumstances [129]. The degree of the biofilm formation ability is strain-specific in *Campylobacter*. For instance, some strains are not able to form biofilms at all, whereas others show biofilm formation at different degrees, suggesting that the genetic composition of the specific strain also plays a role in biofilm formation. Recently, Sung and Khan (2015) and Feng et al. (2016) summarized the molecular mechanisms of *C. jejuni* biofilm formation [130,131]. Briefly, genes involved in motility, flagellar composition (*flaAB*), oxidative stress response, LOS structure composition (e.g., *waaF*, *lgtF*) or cell structure modification (*pgp1*) are involved in biofilm formation. By combining genotyping data and biofilm assays, Pascoe et al. (2015) identified hotspots of genetic variation that corresponded to specific biofilm phenotypes [132]. Various environmental factors influence biofilm formation as well [129]. For example, *Campylobacter* biofilm formation under aerobic conditions is promoted by oxidative stress, presence of organic material or extracellular DNA. On the other hand, biofilm formation is reduced by substances causing osmotic stress. Favorable conditions outside or adverse conditions inside the biofilm can activate biofilm dispersion; for example, the accumulation of signal molecules or increased oxygen levels [133]. Potential biofilm control strategies include modifying the surface of contact structures to reduce attachment and disrupting the biofilm matrix through the application of bacteriophages, bacteriocins, DNase or quorum quenchers (summarized in [134]). Preliminary data are available on the reduction of biofilm mass by application of trans-cinnamaldehyde, eugenol or carvacrol after initial biofilm formation [135]. Zinc oxide nanoparticles, which have a high oxidative potential, can also inhibit biofilm formation [136].

Our knowledge of the role of quorum sensing (QS) mechanisms in *Campylobacter* is still sparse, even though the presence of LuxS and AI-2 production in *C. jejuni* was already demonstrated in 2002 [137]. We still lack description of autoinducer receptors in *Campylobacter*. It is speculated that AI-2 is sensed via a two-component regulatory system [138]. There is no clear picture on the role of *luxS* in *C. jejuni*. LuxS mutants showed a reduction in growth, motility, biofilm formation, reaction to oxygen stress, adhesion/invasion, and colonization. However, these phenotypes varied considerably between respective studies. Different substances have already been tested for their potential to disrupt QS mechanisms (quorum quenching). Simunovic et al. (2020) tested 20 plant extracts, almost all of which altered QS-related phenotypes of *C. jejuni* [139]. For instance, citrus extracts lowered AI-2 activity and influenced motility. As mentioned above, adhesion and invasion in cell cultures, expression of virulence factors (*cadF*, *ciaB*) and biofilm development were also impaired [62,140].

As described for other bacteria, *Campylobacter* is capable of transitioning into a viable but non-culturable (VBNC) state under stress conditions, such as aerobic atmosphere, acid stress, starvation or prolonged cold exposure [83]. Notably, *Campylobacter* in VBNC state exhibit higher resistance to disinfecting agents, initiate biofilm formation and are still infectious.

## 5. Anti-Microbial and Anti-Inflammatory Therapy of Campylobacteriosis

The value of molecular targets enabling modulation of inflammation in *C. jejuni* infections is supported by the fact that antibiotics are not indicated to mitigate enteritis symptoms. The intestinal environment reduces antibiotic efficacy by absorptive removal and diarrheal dilution, both favoring resistance development of the pathogen [141]. In addition, inadequate and improper use of antibiotics has increased *C. jejuni* resistance to macrolides and fluoroquinolones. Both antibiotics were originally reserved for treatment of particularly severe enteritis with systemic manifestations [142]. In consequence, patients receive symptomatic therapies, including rehydration and electrolyte substitution, without causative measures and need to sustain symptoms of intestinal inflammation, which in turn elevates the risk of post-infectious sequelae of infection as outlined above. 

Studies of the mechanisms used by *C. jejuni* to cause intestinal inflammation revealed that the bacteria lack or at least do not rely on exotoxins that are typically produced by other enteric pathogens. Thus, the majority of *C. jejuni* strains produce neither cholera-like enterotoxin nor CDT [12,13,143,144]. Instead, endotoxins play a major role in the immunopathogenesis of campylobacteriosis. In gastroenterology, campylobacteriosis is perceived as an inflammatory syndrome caused by innate immune cells, such as neutrophilic granulocytes and macrophages, which are activated by direct contact with *C. jejuni* (reviewed by [145,146]). In this regard, campylobacteriosis shares common features with the acute purulent *Neisseria meningitidis* and *Neisseria gonorrhoeae* infections affecting other body compartments [147,148]. Early studies on the histology of *C. jejuni* infection proved that neutrophils and macrophages accumulate at intestinal sites of *C. jejuni* invasion [11,147,149]. Upon activation by bacterial endotoxins, these cells trigger inflammation and tissue destruction by the production of pro-inflammatory mediators and reactive oxygen species (ROS). In this scenario, it is tempting to speculate that ROS plays a key role in induction of intestinal apoptosis. This assumption was confirmed by clinical studies, showing that both severity of campylobacteriosis and post-infectious sequelae are significantly associated with distinct LOS variants produced by the infecting *C. jejuni* strains [15,150]. In addition, LOS was shown to be a master regulator of pathogenesis, triggering inflammation, apoptosis, tissue destruction and diarrhea via active induction of sodium malabsorption [150]. Structural variability of LOS has been further shown to be responsible for the heterogeneity of disease symptoms. Hence, innate immune activation by *C. jejuni* endotoxins is key to the treatment of campylobacteriosis and prophylaxis of post-infectious sequelae (reviewed by [145]). Both the LOS and respective immune responses induced via its detection by Toll-like-receptor (TLR) 4 serve as potent molecular targets for dampening inflammation and ameliorating campylobacteriosis [145]. 

Based on these findings, a combination of anti-microbial and anti-inflammatory drugs would represent an ideal treatment option for human campylobacteriosis. Indeed, this conclusion was further supported by results from novel murine infection models that displayed the symptoms as well as the molecular immunopathogenesis induced by *C. jejuni* infections in humans. Notably, mice showed colonization resistance to *C. jejuni*, as well as a high tolerance to LOS, mainly due to the fact that the murine TLR4 responses are approximately 10,000-fold weaker [151,152] compared to humans [153]. Hence, the development of suitable murine infection models required modification of both the intestinal microbiota composition and the LOS responses of mice [145,154,155]. Thus, the major role of *C. jejuni* LOS in human campylobacteriosis was confirmed by manipulation of the murine immune system. Mice with deficiencies in IL-10 [156], single IgG IL-1 Related Receptor (SIGIRR) [154,157] and mice subjected to zinc depletion [158] developed campylobacteriosis symptoms upon *C. jejuni* infection. Since IL-10 [159,160,161] and SIGIRR [154] signaling pathways, as well as zinc application [162,163,164], effectively suppress LOS and lipo-polysaccharide (LPS)-mediated inflammation, *C. jejuni*-induced disease in mice is mainly caused by enhanced pro-inflammatory immune responses to enteropathogenic LOS [145]. Due to its suppressive role in endotoxin signaling, oral zinc supplementation is used to protect children in low-income countries from bacterial diarrheal diseases, including campylobacteriosis [165]. Hence, the development of novel murine infection models represents a breakthrough in *Campylobacter* research and helped to identify LOS-induced inflammation as an innovative molecular target for amelioration of campylobacteriosis in infected humans [145]. 

In particular, secondary abiotic (SAB) IL-10-deficient mice have proven useful for the analysis of *C. jejuni*–host interactions, mainly because the immunopathology characterized by granulocyte and macrophage recruitment, by activation of T and B lymphocytes and by colonic epithelial cell apoptosis mirrors the immune and histopathological responses in *C. jejuni*-infected humans [11,150,166]. In this model, campylobacteriosis depends on the motility and adhesive properties of the pathogen [167]. Most important was the finding that the immunosuppressive drug rapamycin prevented campylobacteriosis in these mice and supported clearing of the pathogen [166], which provided the first experimental proof that the dampening of inflammation is a valuable target for prevention and treatment of *C. jejuni* infection. Furthermore, preclinical placebo-controlled intervention studies on SAB IL-10-deficient mice revealed that defined vitamins, including ascorbate [168] and vitamin D [73,169], the short chain fatty acid butyrate [170] and plant-derived compounds that have been used for an extensive period of time in traditional medicine, such as essential oils [171,172,173,174], curcumin [74], resveratrol [175], carvacrol [176], urolithin-A [177] and activated charcoal [178], effectively dampened inflammation in the course of murine campylobacteriosis. Their respective mechanisms of action demonstrate that (i) LOS activity, (ii) pro-inflammatory mediators including oxygen radicals, (iii) intestinal barrier function and (iv) *C. jejuni*-related pathogenicity factors are well suited as molecular targets for the treatment and prevention of campylobacteriosis [157,179,180]. Finally, based on the key role of iron in ROS formation via the Fenton reaction, it was shown that iron deprivation by desferoxamine ameliorated murine campylobacteriosis [181]. In line with the inactivation of ROS by ascorbate (as mentioned above), these data showed that ROS production by innate immune cells represents another molecular target for treatment of human campylobacteriosis. Thus, progress in murine research provided evidence that traditional medicinal drugs may not only ameliorate human campylobacteriosis, but may also reduce the risk for the appearance of post-infectious sequelae such as GBS or reactive arthritis after LOS-induced inflammation. However, this demands further investigation in murine models of GBS [182,183,184]. Hence, the discovery of the pivotal role of LOS in the induction of acute enteritis and of post-infectious sequelae upon *C. jejuni* infection has greatly contributed to the identification of defined molecules for the treatment and prophylaxis of human campylobacteriosis.

## 6. Concluding Remarks

The early formulated “endotoxin concept” of *C. jejuni*-induced inflammatory diarrhea [148,149] proved valuable, and this has inspired the development of novel murine infection models that are useful for preclinical evaluation of therapeutic and prophylactic strategies to combat human campylobacteriosis. Scientific progress in the understanding of molecular host–pathogen interactions underlying *C. jejuni* colonization, adhesion, invasion and environmental adaptation, and their potential to induce innate immune activation leading to enteritis, have provided molecular targets for innovative treatment options. The principal concept of bacterial LOS playing a pivotal role in the molecular immunopathogenesis of acute campylobacteriosis and its post-infectious sequelae has successfully been used to overcome LOS/LPS tolerance of mice to solve the puzzle of asymptomatic *Campylobacter* colonization in poultry versus acute disease in infected humans. Resistance to LOS is 100-fold to 1,000,000-fold higher in chickens and other birds than in mice and humans, respectively [153,185]. Thus, LOS might help to understand why chickens and other poultry do not develop intestinal inflammation upon *C. jejuni* colonization, as was hypothesized earlier [8]. 

For the future, it is tempting to speculate that combinations of anti-inflammatory and anti-microbial drugs obtained from traditional and conventional medicine will help to dampen both host intestinal inflammation and anti-bacterial resistance (reviewed by [145]). The innovative discoveries summarized here support preventive measures in farming, as well as clinical studies aiming to improve treatment and prophylaxis of human campylobacteriosis.

## Figures and Tables

**Table 1:** Factors influencing survival of *Campylobacter* from the farm to the consumer.

Stage	Factors	References
Environment	UV light, oxygen concentration, dehydration, temperature	[81,82]
Farm	Biosecurity	[83]
	Cleaning and disinfection	[84]
	Vaccination	[85,86]
	Bacteriophage application	[87,88]
	Competitive exclusion	[89]
	Probiotics	[90]
	Bacteriocins	[91]
	Feed supplements (e.g., carvacrol, curcumin, cinnamon oil); drinking water supplements (e.g., organic acids)	[83,92,93]
Slaughter	Scalding temperature	[94,95]
	Hot steam	[96,97]
	Washing or rinsing	[98]
	Chlorinated water	[99]
	Sodium hypochlorite solutions	[100]
	High-pressure spray	[101]
	Cooling	[102]
Food processing	Additives (e.g., organic acids, marinades, spices, sugar)	[103,104,105]
	Modified atmosphere packaging	[106]
	Chilling or freezing	[107]
	Bioactive packaging (immobilized zinc oxide nanoparticles, immobilized bacteriophages)	[108,109]

## Data Availability

Not applicable.
